# Great Toe Strength and Direct Assessment Methods: A Scoping Review for Clinical Applications

**DOI:** 10.3390/bioengineering13080890

**Published:** 2026-07-31

**Authors:** Raghuveer Chandrashekhar, Hongwu Wang, Frank Castro, Jenna Bennett, Jane Morgan-Daniel

**Affiliations:** 1 Human Engineering Research Laboratory (HERL), Department of Veterans Affairs, University of Pittsburgh, Pittsburgh, PA 15260, USA; 2 Department of Occupational Therapy, University of Florida, Gainesville, FL 32611, USA; 3 Department of Liberal Arts and Sciences, University of Florida, Gainesville, FL 32611, USA; frankdanielcastrogonzalez@gmail.com; 4 Food Science and Human Nutrition Department, University of Florida, Gainesville, FL 32611, USA; jenna.bennett@ufl.edu; 5 Health Sciences Library, University of Florida, Gainesville, FL 32611, USA; morgandanie.jane@ufl.edu

**Keywords:** great toe strength, clinical biomarker, hallux

## Abstract

Great toe strength (GTS) is critical for gait, balance, and mobility, yet reliable assessment methods are lacking. This scoping review aims to evaluate and synthesize the direct methods of GTS assessment and their corresponding measurement protocols to guide clinical and research applications. Following the Joanna Briggs Institute methodology, five databases were searched: PubMed, CINAHL, Embase, Web of Science, and IEEE Xplore. All publications on or before 16 December 2024 were included in this review. Studies were included if they quantified isolated great toe strength in human participants using direct measurement tools with non-normalized force outcomes, while exclusion criteria included non-human models, indirect assessments (e.g., electromyography), and multi-digit or dynamic tasks. Following the initial screening of the results, the information from the included articles were extracted into Excel for analyses and synthesis. Two independent reviewers screened titles/abstracts and full texts using Covidence. Conflicts were resolved via consensus or consultation with a third reviewer. We identified 55 studies involving 2821 participants that met our inclusion criteria. Four types of direct GTS assessment methods were identified: dynamometry (56%), manual muscle testing (11%), force transducers (13%), and load cells/strain gauges (18%). Dynamometry was the most common but showed variable reliability due to non-standardized protocols. Healthy individuals exhibited higher GTS (95.01 N) than those with conditions like hallux injury (6.1 N), though plantar fasciitis/plantar heel pain populations showed unexpectedly high GTS (133.27 N). No clear age-related GTS trend emerged, likely due to methodological variability. Less than 11% of studies measured extension strength, highlighting a critical research gap. This review emphasizes the need for standardized protocols to enhance GTS assessment reliability and clinical utility.

## 1. Introduction

The great toe (first metatarsophalangeal joint, 1st-MTJ) plays a critical role in gait, postural stability, and balance [1,2,3,4]. During locomotion, the great toe flexor and extensor muscles generate propulsive forces [5], support foot arch integrity [6], contributes to balance through sensory feedback and dynamic foot stabilization [7,8,9], and ensures adequate ground clearance [10], all of which are essential for functional mobility [11]. Impairments such as hallux limitus/rigidus (restrict great toe movement) [12,13], peripheral neuropathy [14,15], or Charcot–Marie–Tooth disease [16] (a hereditary neuropathy causing muscle weakness) can reduce great toe strength, compromising mobility and increasing fall risks [17,18,19]. Emerging evidence also positions great toe strength (GTS) as a potential biomarker for age- and sex-related health decline, further underscoring its clinical relevance [5,15,20,21,22,23]. The societal burden of GTS impairment is substantial, particularly in aging populations where diminished toe strength contributes to mobility loss, fall-related injuries, and increased healthcare costs associated with fracture rehabilitation and long-term care [7,19].

Despite its diagnostic and prognostic value, reliable GTS measurement remains challenging. Indirect methods (e.g., electromyography) primarily focus on the physiological and electrical activity properties of the muscles but do not quantify force output, making direct methods like dynamometry more suitable for GTS evaluation. However, direct techniques [24], including dynamometry, manual muscle test (MMT), force transducers, and pedobarography, suffer from limitations such as cost, subjectivity, and operational complexity [25,26,27]. For the purposes of this review, dynamometry and force transducers are distinguished based on the device type rather than the measurement principle and this distinction can be attributed to the standardization, calibration, and reproducibility of the device across different studies rather than the difference in the underlying construct, i.e., GTS. Although numerous studies employ these direct methods, there is no consensus on a standardized protocol, critical evaluations of the variation in GTS across age, the different methods and protocols of assessment. While there are reviews focusing on the reliability and challenges of direct and indirect methods of assessment of intrinsic foot muscles [24,28], to the best of our knowledge, there are no known reviews that critically evaluate the direct methods of GTS assessment. Given these challenges, a comprehensive evaluation of direct GTS assessment methods is needed to improve diagnostic accuracy. This scoping review evaluates direct GTS assessment methods to address the lack of standardized protocols and guide their clinical and research applications. Evaluating these direct methods can identify optimal and suboptimal practices for GTS assessment. This could serve as a foundation for standardizing muscle strength measurement of the great toe, and for informing best practices in similar distal muscles and muscle groups.

## 2. Materials and Methods

A preliminary search of systematic review registries (BioMed Central, Cochrane, PROSPERO, etc.) using terms (“big toe” OR “great toe” OR hallux) AND (measure) AND (strength) (27 November 2024) revealed no prior syntheses on this topic, highlighting the novelty and necessity of this work. The protocol for this scoping review was registered on the Open Science Framework (OSF) registry (10.17605/OSF.IO/F6K9X). By synthesizing evidence on the strengths and limitations of current methods, this review aims to guide clinicians and researchers in selecting context-appropriate assessment tools and inform the development of standardized protocols to improve diagnostic accuracy and patient outcomes.

### 2.1. Search Strategy

This scoping review was conducted in accordance with the Joanna Briggs Institute (JBI) methodology for scoping reviews [29]. A three-step search strategy was utilized in this review. First, an initial limited search of MEDLINE (PubMed) and CINAHL (EBSCO) was conducted to identify relevant keywords and index terms (e.g., MeSH, Emtree). Second, using terms derived from the initial search (e.g., “(‘great toe’ OR ‘hallux’) AND (‘strength’ OR ‘force’) AND (‘dynamometry’ OR ‘measurement’)”), a comprehensive search was executed across PubMed, CINAHL, Embase, Web of Science, and IEEE Xplore (to capture engineering/device-focused studies). The search strategy, including all identified keywords and index terms, was adapted for each included database. For example, PubMed was searched using: (‘great toe’ OR hallux) AND (strength OR force) AND (dynamometry OR measurement). The full strategy, including Boolean operators and database-specific adaptations, is detailed in Appendix A. Last, reference lists of included studies were screened to identify additional sources. Studies in English, regardless of publication year, were included. The search was conducted on 16 December 2024, and all publications on or prior to this date were included in this review. Non-English studies and those with unavailable full texts (after contacting corresponding authors twice via email over four weeks) were excluded.

### 2.2. Eligibility Criteria

Guided by the Population, Concept, Context (PCC) framework, inclusion and exclusion criteria were defined as follows:

#### 2.2.1. Population (P)

- Inclusion criteria: human participants of any age or health status.
- Exclusion criteria: non-human studies (e.g., animal models, computational simulations).

#### 2.2.2. Concept (C)

- Inclusion criteria: studies quantifying isolated great toe strength (flexion/extension) using direct measurement tools (e.g., dynamometry, manual muscle testing).
- Exclusion criteria:
  1. Indirect assessments (e.g., electromyography, imaging without force quantification).
  2. Measurements during dynamic activities (e.g., walking, running) or non-isolated tasks (e.g., toe grip dynamometry involving multiple digits).
  3. Pedobarography/pressure mapping without intentional great toe movement (e.g., static standing).
  4. Toe grip dynamometry was excluded to focus on isolated 1st-MTJ strength, as multi-digit tasks obscure specific great toe contributions.

#### 2.2.3. Context (C)

- Inclusion criteria: studies reporting quantitative outcomes of great toe strength (e.g., force in Newtons, torque, or standardized strength scores and not normalized forces, torque, or percentage body weight, etc.).
- Exclusion criteria: qualitative assessments (e.g., screening tests like the paper grip test) or studies omitting numerical results.

#### 2.2.4. Study Selection and Data Extraction

Two independent reviewers screened titles/abstracts and full texts using Covidence [30]. Conflicts were resolved via consensus or consultation with a third reviewer. Data from eligible studies were extracted into a standardized template, including (1) participant demographics (age, health status), (2) measurement methodology (device type, protocol, joint position), (3) outcome metrics (strength values, reliability data), and (4) reported limitations of the method. Study quality was not formally assessed, as per JBI scoping review guidelines, but methodological limitations such as sample size and unclear protocols were extracted and summarized. Following the initial screening of the results, the information from the included articles were extracted into Excel for analyses and synthesis. Articles were deemed irrelevant if they failed to meet any of the inclusion criteria listed within the PCC framework. Additionally, articles were excluded for having the “ineligible outcome metrics” if GTS was reported as a torque, normalized by body weight, or measured using methods listed in the exclusion criteria of “Concept” in the PCC framework. Formal quality assessment of the included studies was not conducted, as this review focuses on characterizing the direct methods and sensor technologies used to assess GTS rather than evaluating treatment efficacy or clinical trial outcomes. Standard quality assessment tools, such as those designed for randomized controlled trials, would not be well suited for evaluating this type of methodological research.

## 3. Results

### 3.1. Study Selection

**Figure 1:**
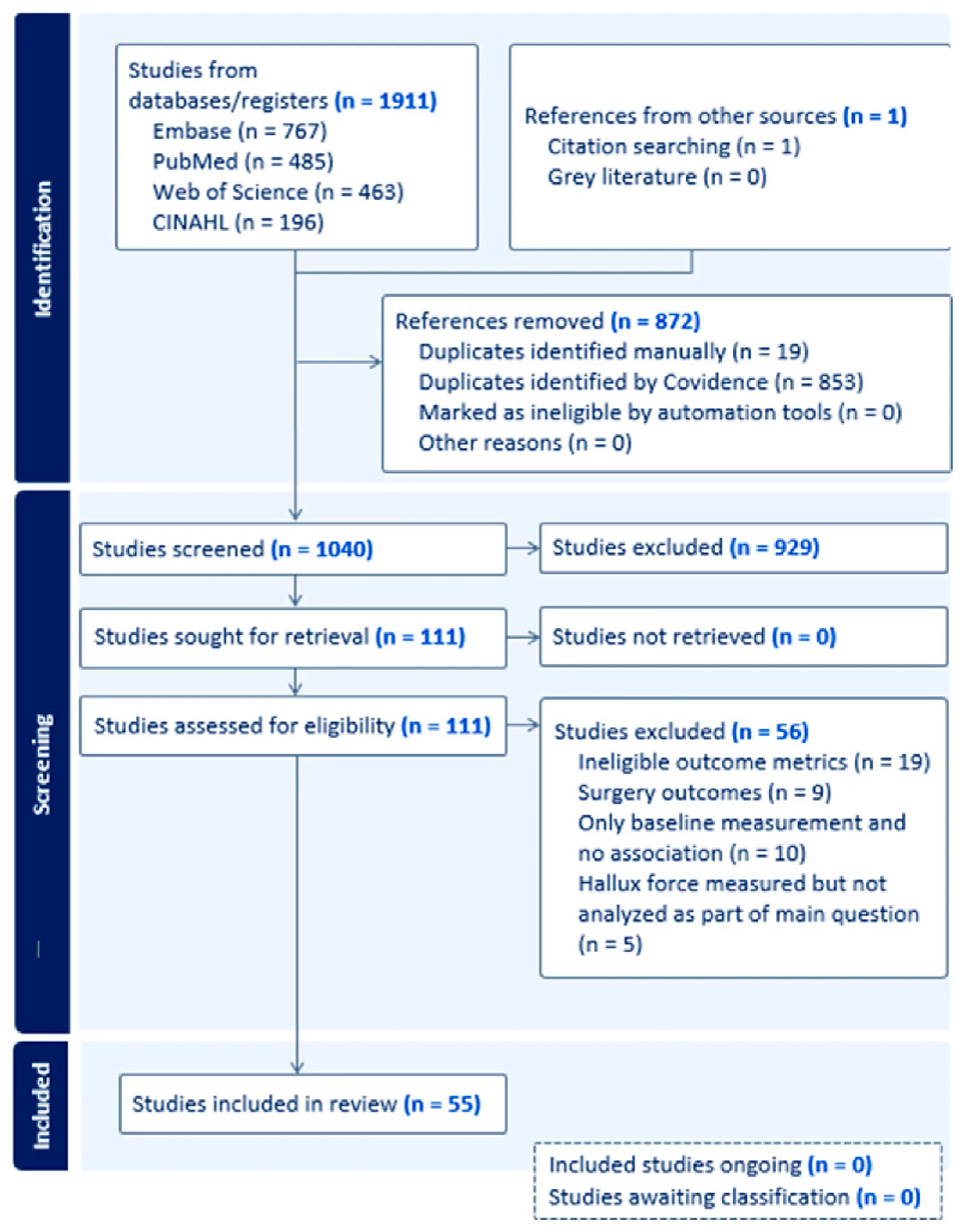
PRISMA flowchart with details regarding the reasons for inclusion/exclusion of the studies based on the eligibility criteria.

The search yielded 1911 articles (CINAHL: 196, Embase: 767, PubMed: 485, and Web of Science: 463). After removing 872 duplicates, 1039 records were screened, and 929 studies were excluded as irrelevant. Full-text review of 110 articles yielded 55 studies (Figure 1). The detailed description of the sample size, health condition/diagnosis, device used, and protocol are all presented in Supplementary Table S1. An additional 30 references were cited in Section 1 and Section 4 to provide background context and support the rationale and interpretation of findings for this review; these 30 studies did not meet the inclusion criteria for this review and were hence not included in the systematic analysis and were not subjected to the same screening and data extraction process.

### 3.2. Assessment Method Characteristics

Of the 55 studies included in this review, four types of GTS assessment were identified: dynamometry, MMT, force transducer/sensor, and load cell/strain gauge. Dynamometry (custom, fixed, or handheld) was the most used method of GTS assessment reported in 56% (32 of 55) [3,12,14,27,31,32,33,34,35,36,37,38,39,40,41,42,43,44,45,46,47,48,49,50,51,52,53,54,55,56,57] of the included studies. Of the other assessment methods identified, 18% of all studies (10 of 55) used load cells or strain gauges [58,59,60,61,62,63,64,65,66,67], 13% (6 of 55) used force transducers or sensors [68,69,70,71,72,73], 11% (6 of 55) used MMT [74,75,76,77,78,79], and one study (2%) mentioned the use of a custom device but did not specify the type of device/sensor used for GTS assessment and it was categorized as not reported (NR) [80]. These different types GTS assessment methods are reported below in Table 1 along with their corresponding reliabilities.

### 3.3. Participant Characteristics

**Figure 2:**
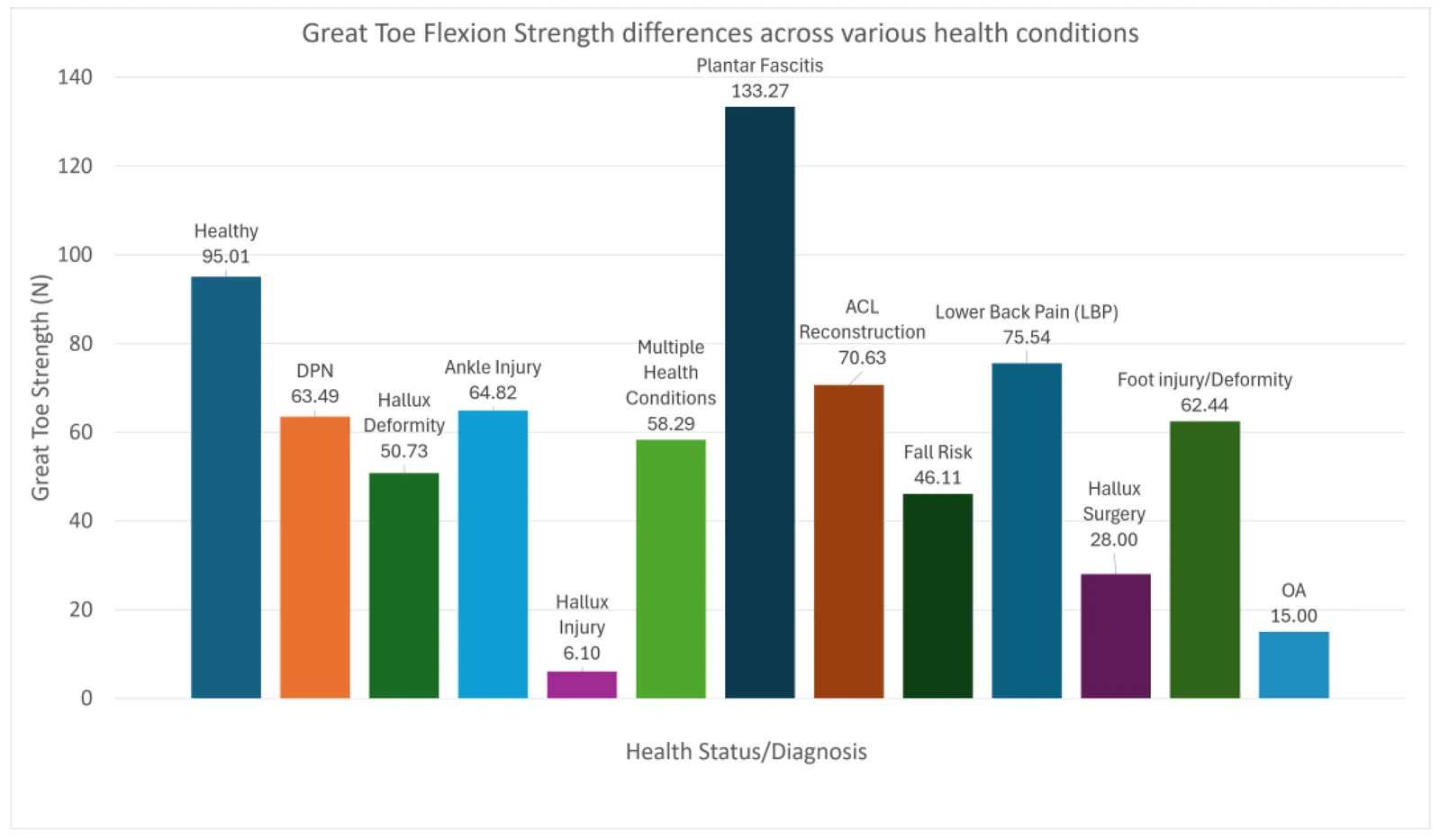
Graphical representation of great toe flexion strength by health status/diagnosis.

The 55 studies included 2821 participants (range: 6–197 N) with various diagnoses. Of the 55 studies included, 19 of the 55 studies, focused on healthy populations [3,27,31,32,34,35,36,40,46,47,56,58,60,63,64,68,70,71,72]. The rest of the studies included participants across various diagnoses that have been organized into seven different categories. The summary of the number of studies, as well as the included participants in each category of the diagnosis, is provided in Table 2, along with the corresponding average great toe flexion force, and represented graphically in Figure 2. The health conditions and diagnoses’ categories are listed in detail in Supplementary Table S2.

### 3.4. GTS and Age

**Figure 3:**
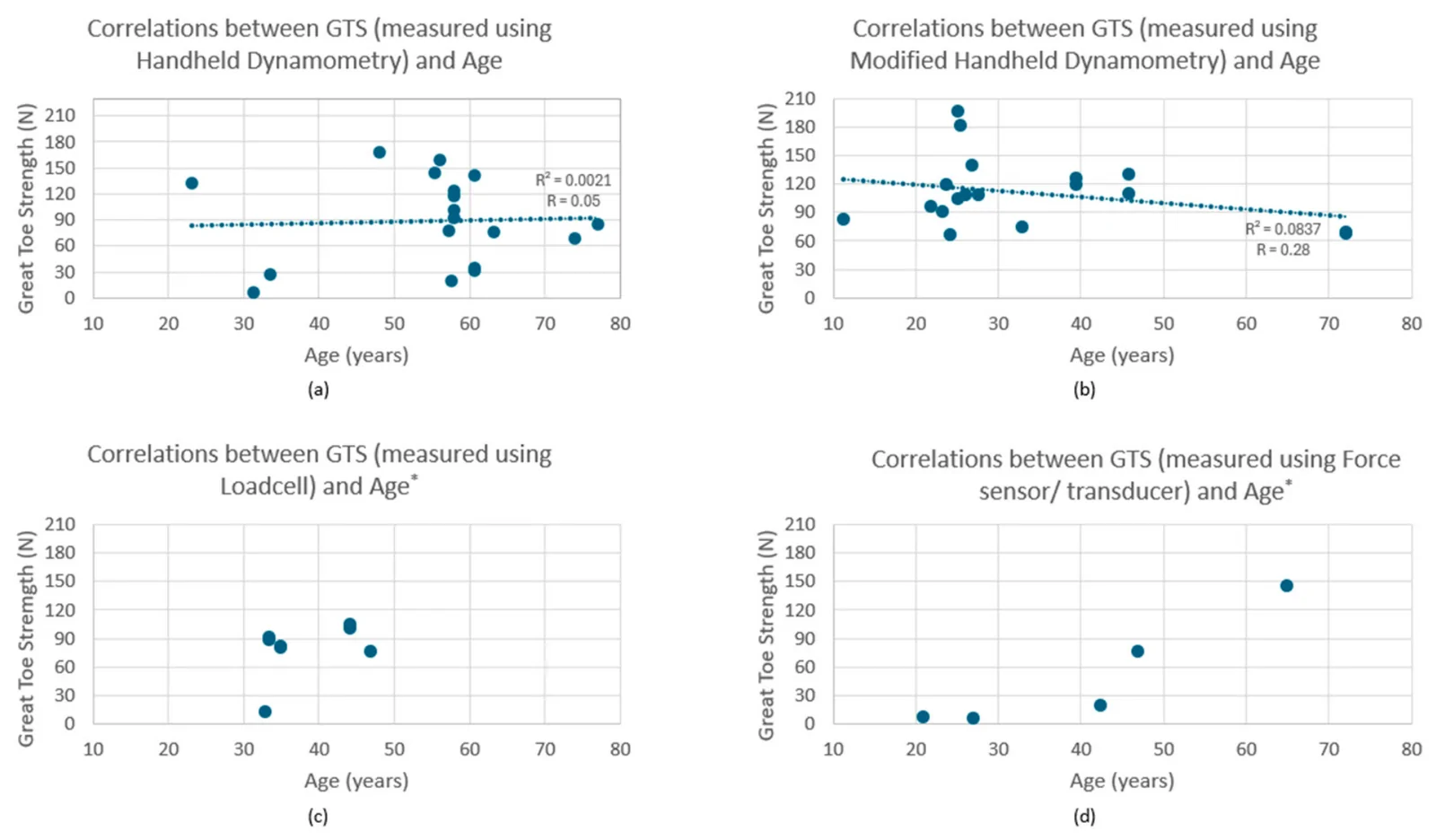
Correlation of GTS with age across different assessment methods reported in the included studies; (**a**) Top left: GTS measured using handheld dynamometry, (**b**) Top right: GTS measured using modified handheld dynamometry, (**c**) Bottom left: GTS measured using a Loadcell, (**d**) GTS measured using a force sensor/transducer; the data presented in the plots include GTS (flexion-only) data of only healthy adults as GTS of individuals with different health conditions could skew the correlation of GTS with age; Each bullet represents a data point (average GTS) and the dashed line represents the trend line *: there were not enough data points for an accurate trendline.

**Figure 4:**
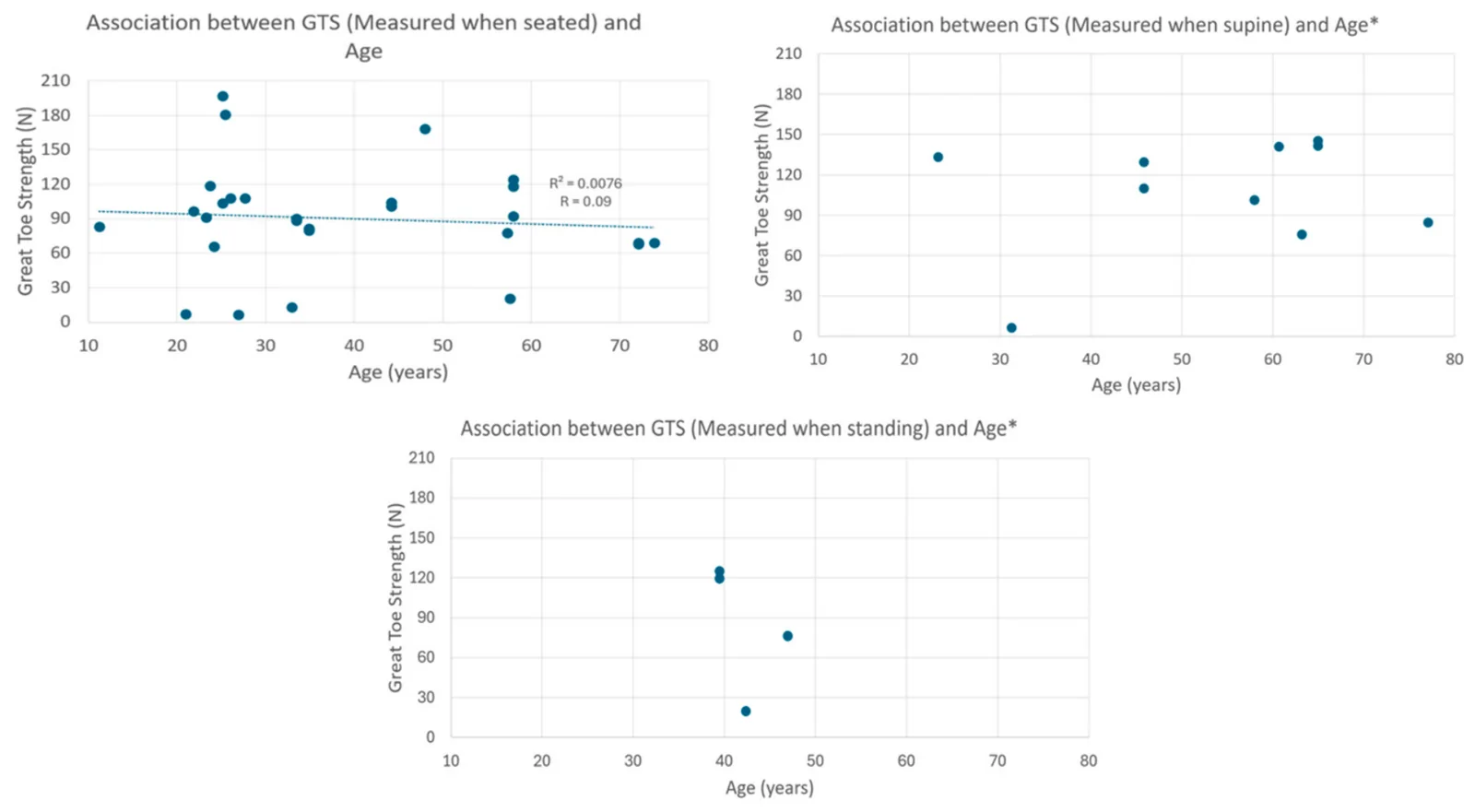
Correlation of GTS with age across different measurement protocols reported in the included studies; Top left: GTS measured in the seated position, Top right: GTS measured in the supine position, Bottom center: GTS measured in standing position; the data presented in the plots include GTS (flexion-only) data of only healthy adults as GTS of individuals with different health conditions could skew the correlation of GTS with age; Each bullet represents a data point (average GTS) and the dashed line represents the trend line *: there were not enough data points for an accurate trendline.

Overall, among all the healthy adults’ sample included in this review (n = 649) no consistent age-related trend emerged, likely due to variability in the type of assessment methods (Figure 3) and/or measurement protocol (Figure 4). However, the highest GTS values were reported among individuals aged between 18 and 29 years (n = 272), with a maximum of 196.53 N. Upon closer evaluation, within individuals aged 30–59 years (n = 296), the maximum reported GTS was 168.10 N and the maximum among individuals aged 60 years and older (n = 81) it was 145.20 N. Fixed dynamometry showed stronger age–GTS correlations than handheld methods.

This inconsistency in assessment methods and/or devices used likely contributed to the variability in the results. Additionally, the variability could also be potentially attributed to the participants’ posture during testing, i.e., seated, supine, or standing, and the plots of GTS across different ages measured in each posture/condition is presented below in Figure 4. The plots in Figure 3 and Figure 4 only included the great toe flexion forces as only six of 55 studies (10.91%) reported great toe extension force, of which only four studies (7.27%) reported the mean age of the participants’ data. As the muscles involved in extension and flexion of the great toe are different, the great toe extension forces were excluded from all plots. This review also included two studies with an exclusive focus on a pediatric subset (n = 15) [34,75] and one study with a pediatric population examined concurrently with an adult population, although the results specific to pediatric participants were not separately reported in this study (n = 50) [36]. In Quinlan et al. [34], the participants were 11.25 ± 0.38 years old with an average GTS of 72.79 and 82.7 N (two trials); Boyce et al. [75] was a case study with a single participant aged 17 years and GTS was measured using MMT; and lastly, Rowley et al. [36] included participants aged between 17.1 and 26.7 years and the average GTS ranged between 90 and 97 N.

## 4. Discussion

This scoping review synthesized methods and protocols for GTS assessment (with a large number of studies assessing only great toe flexion strength). Dynamometry was the most common method, yet the lack of standardized protocols contributed to inconsistent results across studies. This variability underscores the need for robust, validated protocols to ensure reliable GTS measurements. The absence of a clear age-related GTS trend suggests methodological differences rather than a biological pattern.

Thirty-two of the 55 studies included in this review used traditional (14 of 32) or modified (18 of 32) versions of handheld dynamometry for GTS assessment, where the modified versions involved the handheld dynamometer being fixed in place during assessment. While the overall variability in GTS measured using traditional (SD = 49.65 N) and modified (SD = 44.65 N) handheld dynamometry were comparable, the correlation between age and GTS shows a clearer trend (stronger correlation) in studies using a fixed/modified version of handheld dynamometry compared to the traditional handheld dynamometry. The lack of consensus in the results across studies can also potentially be attributed to GTS assessment protocol involving the participants’ postures during the GTS assessments (Figure 4), which included seated (30 of 54), standing (5 of 54), supine positions (8 of 54), and the remaining (11 of 54) did not report the participants’ posture in their assessment protocol. This further emphasizes the need for more robust/standardized protocols for GTS assessment.

Despite the large variability in the GTS across individuals with different health conditions (6.10–133.27 N), the studies included in this review show that healthy individuals had the strongest toe strength when compared to individuals with different health conditions. The average GTS of healthy individuals (95.01 N) was 88.91 N larger than that of individuals with hallux injury (6.10 N) [48] and 80.01 N larger than that of individuals with osteoarthritis (15.00 N) [52]. The one exception in this trend is among individuals with plantar fasciitis/plantar heel pain (133.27 N) [41,42], who had an average GTS of 38.26 N larger than that of healthy individuals. This anomaly can be attributed to one of the two studies categorized in the plantar fasciitis/plantar heel pain group, Barnes et al. [41], involving individuals diagnosed with chronic and recent onset plantar heel pain. The participants in this study, however, were reported to have a normal to healthy hallux range of motion. Additionally, it may be tentatively hypothesized that individuals with heel pain or plantar fasciitis adopt compensatory gait patterns, offloading the painful heel and increasing forefoot loading [81], which could over time strengthen the great toe flexor musculature and may explain the trend toward higher GTS in this group. However, this interpretation warrants caution, as intrinsic foot muscle weakness has also been reported in this population [41,82]. There are three studies that report notably low GTS values compared to other studies with healthy individuals, which can potentially be attributed to the GTS assessment protocol and/or the unclear hardware specifications [58,68,72]. In Nihal et al. [68] the participants were asked to “…lift the hallux up and then press down onto the sensor with as much force as possible for approximately three to five seconds.” and GTS was measured using a FlexiForce^®^, ((Mylar^®^) film Tekscan Inc., Boston, MA, USA) [68]. In Bojsen-Moller et al. [58] the participants’ GTS was measured by taking the average of “…2–3 isometric maximal voluntary contractions by gradually increasing plantarflexion force over a 3 s period from a relaxed state to maximal effort. Each trial was separated by a 2 min rest…” using a small load sensor cell (Noraxon, Inline Flexiforce sensor, Scottsdale, AZ, USA) placed below the distal part of the toe [58]. Lastly, in Gomez-Carrion et al. [72] the participants were asked to complete the Jack’s test, i.e., “…the test involves the passive dorsiflexion of the 1st-MTJP with the individual in a relaxed stance while elevating the height of the medial arc…” using a digital force gauge (FPX^®^25, Wagner Instruments^®^, Greenwich, CT, USA) and this study measured the resistance force by pushing the gauge against a stationary toe, which does not accurately reflect GTS flexion force that could be obtained from voluntary contraction [72]. The technical specifications of the sensors used in both Nihal et al. [68] and Bojsen-Moller et al. [58] were not reported, leaving open the possibility that sensors with a relatively low maximum load capacity were utilized. This possibility should be considered when interpreting their results, particularly in the context of muscle strength assessments. Additionally, unlike the other studies, these protocols differed in that the force measured either the resistance or involved movement of the big toe in a way that minimized the continuous contact between the sensor and the big toe.

One of the key takeaways from this scoping review with respect to the foot posture/protocol of GTS assessment, regardless of being in a seated, supine, or standing position, is to position the foot, more particularly the great toe, in the anatomical neutral position. Fourteen of the 55 studies explicitly state maintaining the foot and/or the hallux in the anatomically neutral position during the GTS measurement. Only seven of the 55 studies included in this review measured GTS with the foot and/or great toe in a non-neutral position. Of the remaining 36 studies, 11 were those that did not report foot posture in their protocol and the other 25 studies mention the hip, knee, ankle, and/or toe angles during the measurement but do not explicitly state that the posture was akin to the anatomical neutral position. Furthermore, there are a couple of other studies that report the importance of maintaining the foot and great toe in the anatomically neutral position to prevent the influence of other muscles in the intrinsic foot muscle complex and help isolate the great toe during GTS assessments [83,84]. Lastly, another significant finding in this review is the critical gap in the literature regarding great toe extension strength (GTES) as <11% of the studies reported GTES. Great toe extension is biomechanically crucial for activating the windlass mechanism, which helps transform the foot into a rigid lever for push-off during gait. Great toe extension also plays an important role in shock absorption, adapting the shape of the foot for different terrains, and foot clearance during gait. Additionally, GTES weakness has been associated with neurological conditions such as neuropathy [85], thus underscoring the urgent need to close this research gap in GTES assessment. Overall, this scoping review highlights the need for better methods for GTS assessment, particularly GTES and its importance as a part of routine clinical practice across individuals with different health conditions. Establishing robust, standardized GTS assessment would improve measurement accuracy and, in turn, the standard of care for conditions such as neuropathy, hallux valgus, and toe deformities, which are linked to balance, mobility, and increased fall risk.

## 5. Limitations

This scoping review has several limitations that should be considered when interpreting its findings. The inclusion of only four studies on great toe extension strength limits the generalizability of the results and may not fully capture the breadth of GTS extension assessment methods. The exclusion of non-English studies may introduce language bias, potentially omitting relevant research from diverse populations. Additionally, the focus on direct measurement methods excluded indirect approaches, such as electromyography, which could provide complementary insights into GTS assessment. Variability in study populations, protocols, and outcome metrics further hindered direct comparisons of the average GTS values, contributing to inconsistent findings, particularly regarding age-related trends. Finally, the reliance on published studies may miss emerging, unpublished innovations in GTS measurement devices, limiting the review’s scope in capturing cutting-edge technologies.

## 6. Conclusions

This scoping review underscores the need for standardized GTS assessment protocols, particularly using fixed dynamometry in the anatomical neutral position to isolate great toe strength. Only <11% of studies measured extension strength, highlighting a critical research gap. Standardized assessments could enhance clinical management of conditions like hallux valgus, peripheral neuropathy, and reduce fall risk in older adults. Future studies should establish normative GTS values and develop standardized protocols for diverse populations to potentially improve diagnostic accuracy and patient outcomes.

## Figures and Tables

**Table 1:** Summary of the types of methods/sensors used for GTS assessments.

GTS Assessment Method (Device/Sensor)	Number of Studies	Reliability
Dynamometer	32	(0.77–0.95) ^$^,(0.87–0.97) ^$^ [31]; 0.93 ^$^ [34]; (0.803–0.962) ^^,#,$^ [35]; >0.75 ^$^ [36]; 0.923 ^$^, 0.938 ^$^ [37]; (0.81–0.92) ^^^, (0.72–0.96) ^#^ [27]; 0.894 ^$^ [40]; (0.807–0.913) ^#^ [42]; (0.842–0.952) ^^,#,$^ [64]; 0.75 ^$^ [46]; 0.874 ^$^ [47]; 0.971 ^^^, 0.992–0.998 ^#^ [72]; 0.938 (0.846–0.968) ^^,#^ [50]; 0.89 ^#^ [53]; 0.848 ^$^ [56];
Manual muscle test (MMT)	6	(0.82–0.99) ^$^ [74]
Force transducer	6	NR
Load cell/strain gauge	10	0.75 ^#^ [59]; 0.982 ^#^ [60]
Not reported	1	NR [80]
Total	55	

^^^: inter-rater reliability; ^#^: intra-rater reliability; ^$^: test–retest reliability; NR: not reported.

**Table 2:** Summary of the number of articles and the corresponding sample distribution by health condition or diagnoses, along with their average great toe flexion strength; only the individuals in the sample from the included studies were used for reporting the average GTS.

Diagnosis	No. of Studies	Total Sample	Included Studies	Included Sample	GTS (N) Mean (SD)	Range (N) (Min–Max)
Healthy	19	757	27 *	950 *	95.01 (47.36)	6–196.53
Diabetic peripheral neuropathy (DPN) ^^^	4	338	3	189	63.49 (41.09)	27.27–124
Hallux deformity	11	447	8	319	50.73 (15.47)	18.64–80.10
Ankle injury	3	79	3	67	64.82 (66.85)	13.7–169.6
Hallux injury	3	15	1	13	6.1	NA
Multiple health conditions	3	543	3	543	58.29 (33.50)	25.51–114.80
Plantar fasciitis/heel pain	3	427	3	357	133.27 (43.49)	77.49–159.30
ACL reconstruction	2	38	1	37	70.63	NA
Fall risk	1	30	1	15	44.15 (2.77)	42.18–46.11
Lower back pain (LBP)	1	52	1	52	75.53	NA
Spinal Cord Injury (SCI)	1	22	0	0	NA	NA
Hallux Surgery	1	32	1	32	28	NA
Lumbosacral radiculopathy	1	4	0	0	NA	NA
Foot injury/deformity	1	28	1	28	62.44 (0.21)	62.29–62.59
Osteoarthritis (OA)	1	9	1	9	20.1	NA
Total	55	2821	54	2287		

^^^: this indicates a category/diagnosis where some studies did not report an average value but median values of toe strength and were excluded from the calculation of the mean toe strength; *: this indicates that healthy controls from other studies focusing on specific diagnoses have been included within the sample and mean GTS calculation; NA—not applicable.

## Data Availability

The raw data supporting the conclusions of this article will be made available by the authors on request.

## Supplementary Materials

The following supporting information can be downloaded at: https://www.mdpi.com/article/10.3390/bioengineering13080890/s1, Table S1: The detailed description of the sample size, age, health condition/diagnosis, device used, and protocol are all presented in detail; Table S2: The health conditions/diagnoses’ categories are listed in detail using the paper numbers listed in Supplementary Table S1.

## Abbreviations

The following abbreviations are used in this manuscript:

GTS	Great Toe Strength
GTES	Great Toe Extension Strength
GTFS	Great Toe Flexion Strength
PCC	Population, Concept, Context
LBP	Lower Back Pain
SCI	Spinal Cord Injury
DPN	Diabetic Peripheral Neuropathy
MMT	Manual Muscle Test
OA	Osteoarthritis
1st-MTJ	First Metatarsophalangeal Joint

## Appendix A

SEARCH STRATEGY
Databases: PubMed, CINAHL, IEEE, Embase, and Web of Science
1. PUBMED—Total 485
Great Toe—“Hallux”[Mesh] OR “Hallux” OR “big toe” OR “big toes” or “great toe” or “great toes”
(“Hallux”[Title/Abstract] OR “big toe”[Title/Abstract] OR “big toes”[Title/Abstract] OR “great toe”[Title/Abstract] OR “great toes”[Title/Abstract]) OR (“Hallux”[Mesh])
AND
Strength—((strength* OR strong OR force* OR weakness OR power OR weak OR “manual muscle test*” OR dynamomet* OR loadcell) OR (“Muscle Strength”[Mesh:NoExp] OR “Muscle Strength Dynamometer”[Mesh]))
AND
Muscle—((flexor* OR flexion OR dorsiflexion OR plantarflexion OR extensor* OR extension OR muscle*) OR (“Muscle Strength”[Mesh:NoExp] OR “Muscle Strength Dynamometer”[Mesh] OR “Hallux Rigidus”[Mesh] OR “Peroneal Neuropathies”[Mesh] OR “Hallux Limitus”[Mesh]))
AND
English
2. Web of Science—Total 463
Searched in the Topic Field
Great Toe—“Hallux” OR “big toe” OR “big toes” or “great toe” or “great toes”
AND
Strength—strength* OR strong OR force* OR weakness OR power OR weak OR “manual muscle test*” OR dynamomet* OR loadcell
AND
Muscle—flexor* OR flexion OR dorsiflexion OR plantarflexion OR extensor* OR extension OR muscle*
AND
English
3. CINAHL—Total 197
Great Toe—“Hallux” OR “big toe” OR “big toes” or “great toe” or “great toes” OR (MH “Hallux Limitus”) OR (MH “Hallux Rigidus”) OR (MH “Hallux Valgus”)
AND
Strength—strength* OR strong OR force* OR weakness OR power OR weak OR “manual muscle test*” OR dynamomet* OR loadcell OR (MH “Muscle Strength”) OR (MH “Dynamometry”)
AND
Muscle—flexor* OR flexion OR dorsiflexion OR plantarflexion OR extensor* OR extension OR muscle* OR (MH “Flexion+”) OR (MH “Extension+”) OR (MH “Flexor Hallucis Longus”) OR (MH “Muscle Strength”)
AND
English
4. Embase—Total—768
Great Toe—“Hallux” OR “big toe” OR “big toes” or “great toe” or “great toes” OR ‘hallux’/exp OR ‘hallux valgus’/exp OR ‘hallux rigidus’/exp
AND
Strength—strength* OR strong OR force* OR weakness OR power OR weak OR “manual muscle test*” OR dynamomet* OR loadcell OR ‘muscle strength’/exp OR ‘dynamometer’/de OR ‘back/leg/chest dynamometer’/de OR ‘hand dynamometer’/de
AND
Muscle—flexor* OR flexion OR dorsiflexion OR plantarflexion OR extensor* OR extension OR muscle* OR ‘muscle strength’/exp OR ‘flexor hallucis longus muscle’/exp OR ‘flexor hallucis brevis muscle’/exp OR ‘extensor hallucis longus muscle’/exp
AND
English
5. IEEE Xplore—Total 0
Ran search with “All metadata”, when ran with title and abstract, combining searches was not allowed. 9 articles for toe and strength combination—but none of the articles seemed relevant based on inclusion/exclusion criteria.
Great Toe—“Hallux” OR “big toe” OR “big toes” or “great toe” or “great toes”
AND
Strength—strength* OR strong OR force* OR weakness OR power OR weak OR “manual muscle test*” OR dynamomet* OR loadcell
AND
Muscle—flexor* OR flexion OR dorsiflexion OR plantarflexion OR extensor* OR extension OR muscle*
AND
English
